# LC-1000 Flow Cytometry System Improves Risk Stratification of Thyroid Nodules with Suspected Follicular Neoplasm

**DOI:** 10.31662/jmaj.2021-0050

**Published:** 2021-12-24

**Authors:** Ayana Suzuki, Mitsuyoshi Hirokawa, Mitsuru Furutate, Yasuo Hirai, Akira Miyauchi

**Affiliations:** 1 Department of Diagnostic Pathology and Cytology, Kuma Hospital, Kobe, Japan; 2 PCL Japan Pathology and Cytology Center PCL Inc., Kawagoe, Japan; 3 Department of Ob & Gyn., Faculty of Medicine, Dokkyo Medical University, Mibu, Japan; 4 Department of Surgery, Kuma Hospital, Kobe, Japan

**Keywords:** Thyroid, Aspiration cytology, Follicular neoplasm, LC-1000 flow cytometry system, Cell proliferation index, CPIx

For thyroid nodules classified as follicular neoplasm/suspicious for follicular neoplasm (FN/SFN), molecular testing can help with risk assessment ^(1)^. However, in Japan, molecular testing of aspirated materials is not performed because it is expensive and not covered by medical insurance. Thus, the decision to undergo surgery with FN/SFN considers other findings, such as the tumor size, the serum thyroglobulin level, ultrasound findings, tumor expansion into the mediastinum, autonomous functioning, and surrounding tissue compression ^(2)^.

The LC-1000 (Sysmex, Kobe, Japan) is an exfoliative cell analyzer that uses flow cytometry (FCM) to analyze the nuclear DNA ^(3), (4)^. The amount of nuclear DNA correlates with cell proliferation, and increased nuclear DNA levels indicate malignancy ^(3), (4)^. To our knowledge, this is the first report on using the LC-1000 FCM for materials aspirated from the thyroid nodules. This study aimed to evaluate the utility of LC-1000 FCM in the preoperative risk stratification of FN/SFN.

The study protocol was reviewed and approved by the Institutional Review Board of Kuma Hospital (20200213-3). It complied with the 1964 Declaration of Helsinki and its amendments or comparable ethical standards. All study participants provided informed consent. Out of the 1,179 thyroid lesions surgically resected at Kuma Hospital from October 2018 to July 2019, 87 (7.4%) lesions had been classified as “FN/SFN” by a thyroid fine-needle aspiration cytology (FNAC). Among the classified lesions, 17 solitary nodules that were large enough to puncture or suspected to be malignant by ultrasonography were collected for this study. The histological diagnoses included four follicular adenomas (FAs, including one oxyphilic cell variant and one FA with bizarre nuclei), three follicular tumors of uncertain malignant potential (FT-UMPs), one well-differentiated tumor of uncertain malignant potential (WDT-UMP), seven follicular carcinomas (FCs, including four minimally invasive and three widely invasive), one papillary thyroid carcinoma (PTC) with a predominant follicular pattern, and one poorly differentiated thyroid carcinoma (PDTC). The samples were obtained from the resected ex vivo thyroid nodules by aspiration using 22-gage needles. The samples were rinsed in a CelVerse™ Solution (Sysmex, Kobe, Japan) and analyzed by the LC-1000 system.

The LC-1000 consists of two main components: a pretreatment unit and an FCM unit. The former performs cell dispersion and adjusts input cell numbers and cell concentration. The latter performs DNA staining and FCM measurements. We measured the cellular and the nuclear sizes and selected tumor cells by gating (Figure 1a). Subsequently, we created a DNA histogram, and the histogram revealed the presence of two main groups with either a low or a high amount of DNA (Figure 1b). The low DNA group (Group I) was consistent with the G0/G1 phase, and the high DNA group (Group II) was consistent with the S and G2/M phases. Using this information, we calculated the cell proliferation index (Group II/Group I). Statistical analyses were performed using unpaired t-tests and Fisher’s exact test. A P-value of <0.05 was considered statistically significant.

**Figure 1.**
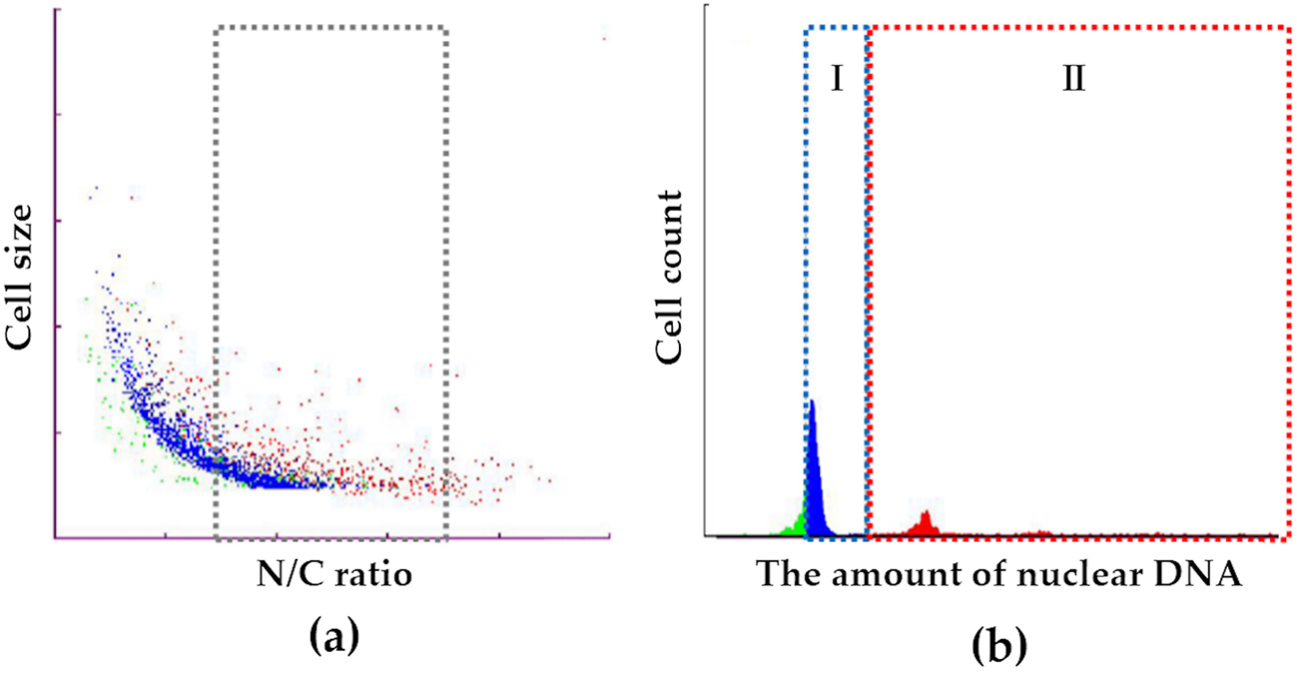
Schematic diagram of the LC-1000 flow cytometry procedure. Cells with a moderate nuclear/cytoplasmic (N/C) ratio were selected as tumor cells, highlighted by the dotted box (a). The tumor cells were classified into two groups, low- (I) and high- (II) DNA amount groups (b).

Cell proliferation index (CPIx) values ranged from 0.08 to 3.00 with a mean of 0.57. CPIx values of FAs, FT-UMPs, WDT-UMP, FCs, PTC, and PDTC were 0.08 to 0.40 (mean = 0.19), 0.30 to 1.5 (mean = 0.72), 0.14, 0.18 to 3.00 (mean = 0.95), 0.22, and 0.61, respectively. The CPIx of FA with bizarre nuclei (0.08) was lower than that of conventional FAs (1.14 and 0.4). CPIx values of FCs tended to be higher than those of FAs, but this trend was not statistically significant. Mean CPIx values of minimally and widely invasive FCs were 1.35 and 0.42, respectively.

Table 1 shows the CPIx values in three categories: benign (FA), intermediate (FT-UMP and WDT-UMP), and malignant (FC, PTC, and PDTC); mean CPIx values were 0.19, 0.58, and 0.83, respectively. The mean CPIx values of the malignant category were higher than benign or intermediate values, reflecting their risk stratification. However, this trend was not statistically significant. We considered CPIx ≥0.16 a positive score. With this cutoff value, the positive rate (100%) in the malignant category was significantly higher than the positive rate (25.0%) in the benign category. One of four FAs had a CPIx ≥0.16. The sensitivity and specificity were 100% and 75.0%, respectively.

**Table 1.**
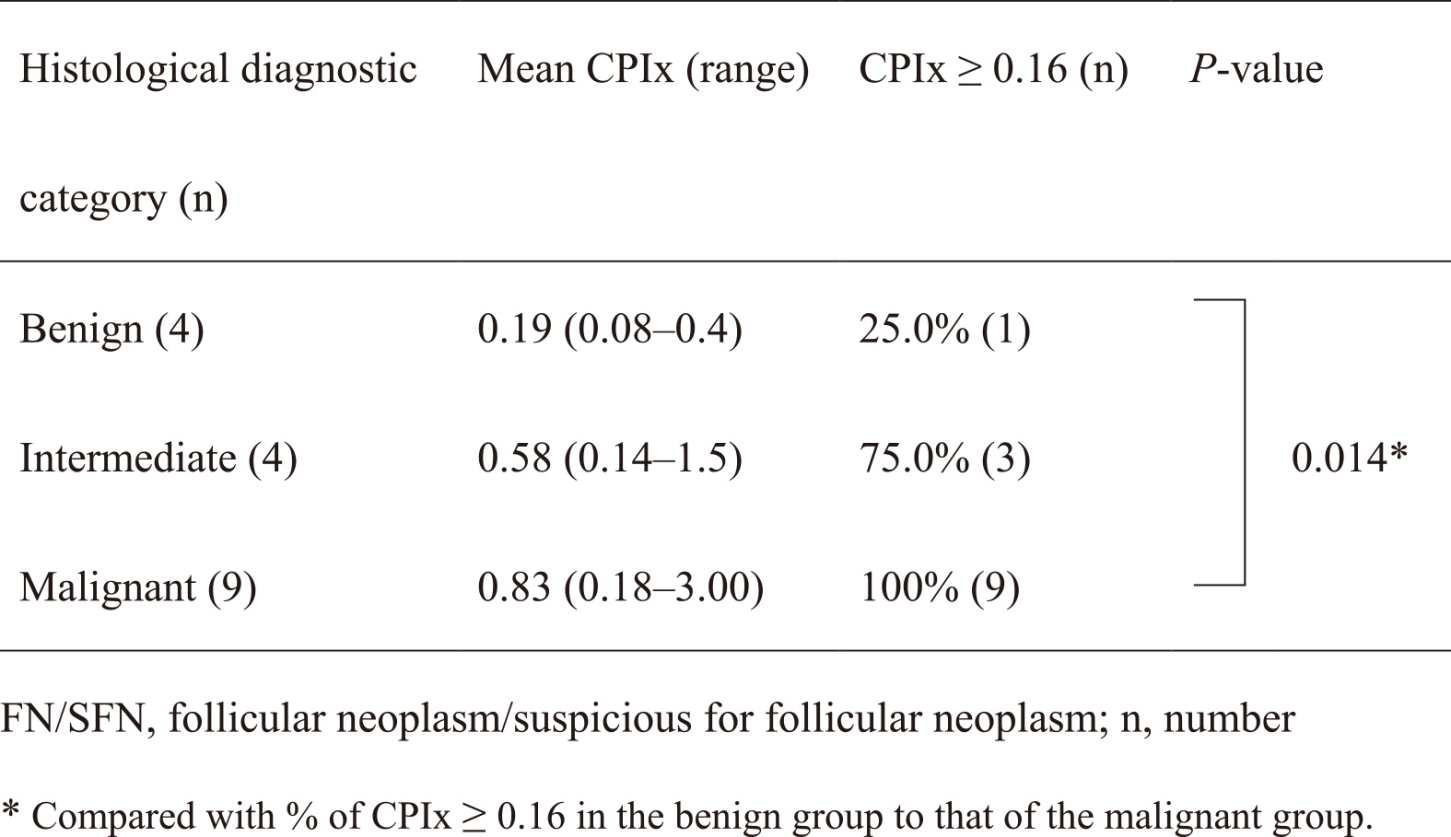
Cell Proliferation Index (CPIx) Values of Thyroid Nodules with FN/SFN Using LC-1000 Flow Cytometry.

Various ancillary techniques to improve diagnoses of thyroid nodules with indeterminate cytology have been proposed throughout the years. Pujani et al. reported Ki-67 as a proliferative marker by demonstrating that Ki-67 labeling indices of the FCs were significantly higher than those of the FAs (mean = 6.00 versus 1.01, respectively) ^(5)^. Furthermore, Mase et al. reported that the immunopositivity for HBME-1 occurred more frequently in FC (84.6%) than in FA (27.4%) ^(6)^. In another study, Borup et al. concluded that down-regulation of factors involved in growth arrest and apoptosis may represent a decisive step in the pathogenesis of FC ^(7)^. However, these factors are impractical for use in FNAC because of inter-observer variation. Recently, molecular testing, which has a high negative predictive value for ruling out cancer, has been applied as an option for the thyroid nodules with indeterminate cytology in Western countries ^(1), (8)^. In Japan, this testing is expensive and not covered by insurance. A similar test that is not cost-prohibitive is critically needed.

FCM provides a rapid multi-parametric analysis of single cells in solution to diagnose and monitor hematologic neoplasms. For thyroid FNAC, CD45-gating FCM is becoming increasingly popular for the lymphoma-suspected lesions ^(9)^. For follicular neoplasms, FCM has been used with the formalin-fixed, paraffin-embedded thyroid tissues ^(10), (11)^. Mattfeldt et al. described that the percentage of cells in the G2/M phase in the FC group was more than twice as high as that in the FA group ^(10)^. Oyama et al. showed a trend toward an increasing aneuploidy from FA to FC, but concluded that there was a limited diagnostic usefulness ^(11)^.

In the present study, the amount of the nuclear DNA for materials aspirated from the FN/SFN nodules was analyzed using the LC-1000 FCM. To increase the reliability of the results, we selected tumor cells and excluded the inflammatory and the non-neoplastic follicular cells by gating. Thus, the CPIx reflects the percentage of cells in the G2/M phase. The CPIx values of the FCs tended to be higher than those of the FAs. Using a CPIx cutoff of 0.16 showed high sensitivity and specificity for detecting the malignant nodules. The results were almost equivalent to those of the molecular tests ^(8)^. From our results, one of the four FAs with a positive CPIx could develop into an FC.

According to our findings, the FN/SFN nodules with a negative CPIx may be a useful indicator that can be followed without surgical resection. Additionally, we have demonstrated that the LC-1000 FCM could be useful in managing the FN/SFN nodules. However, our study has some limitations. The samples used in this study were obtained from the resected thyroid nodules preoperatively reported as FN/SFN, and the sample size was small. A larger case study using preoperative FNAC materials will be necessary to confirm the usefulness of the LC-1000 FCM in the management of the FN/SFN nodules.

## Article Information

### Conflicts of Interest

None

### Acknowledgement

We would like to thank Editage (www.editage.jp) for English language editing.

### Author Contributions

All authors contributed to the study’s conception and design. Ayana Suzuki, Mitsuru Furutate, and Yasuo Hirai performed material preparation, data collection, and analysis. Ayana Suzuki and Mitsuyoshi Hirokawa wrote the first draft of the manuscript, and all authors commented on previous versions of the manuscript. All authors read and approved the final manuscript.

### Approval by Institutional Review Board (IRB)

20200213-3 approved by the Institutional Review Board of Kuma Hospital
